# Prognostic Impact of Nucleophosmin 1 (NPM1) Gene Mutations in Egyptian Acute Myeloid Leukemia Patients

**DOI:** 10.4274/Tjh.2012.0048

**Published:** 2013-06-05

**Authors:** Magda Zidan, Howyda Shaaban, Doaa El Ghannam

**Affiliations:** 1 Banha University Faculty of Medicine, Department of Clinical Pathology, Benha, Egypt; 2 Mansoura University Faculty of Medicine, Department of Clinical Pathology, Mansoura, Egypt

**Keywords:** Myeloid leukemia, NPM1 mutation

## Abstract

**Objective:** Somatic mutations of the nucleophosmin gene (NPM1), which alter the subcellular localization of the product, are the most frequent mutations in patients with acute myeloid leukemia. The aim of the study was to assess the prevalence and prognostic impact of NPM1 gene mutations in adult AML patients.

**Materials and Methods:** Polymerase chain reaction and single-strand conformation polymorphism (PCR-SSCP) were used to screen 55 AML patients for mutations of NPM1 gene.

**Results:** NPM1 mutations were found in 12 (21.8%) of 55 patients, significantly associated with higher total leukocytie count, marrow blast percentage (p=0.03 and p=0.02, respectively), and M5 subtype (p<0.001). Patients with NPM1 mutations had significantly higher complete remission rates (p=0.003) and a trend to lower rates of mortality, relapse and refractory disease (p=0.28, p=0.45 and p=0.08, respectively). Survival analysis showed significantly longer disease-free survival (mean 18.635±1.229 versus 11.041±1.250 months, p=0.044) and overall survival (mean 19.810±1.624 versus 12.063±1.244 months, p=0.041) in patients with NPM1 mutations compared with those without. Multivariate analyses confirmed NPM1 mutation as a significant independent predictor for disease-free survival (HR=0.066, p=0.001) and overall survival (HR=0.125, p=0.002).

**Conclusion:** NPM1 mutation is a prognostic factor for a favorable outcome in Egyptian population. This finding is of major clinical importance since it strongly suggests that NPM1 mutations may allow one to divide the heterogeneous patient group of AML into prognostically different subgroups.

**Conflict of interest:**None declared.

## Abstract (tr)

**Amaç:** Nükleofosmin 1 (NPM1) genine ait olan ve ürünü hücrealtı düzeyde değiştiren somatik mutasyonlar, akut miyeloid lösemi (AML) hastalarında en sık tespit edilen mutasyonlardır. Bu çalışmada AML hastalarında NPM1 gen mutasyonlarının prevalansının ve prognostik öneminin değerlendirilmesi amaçlanmıştır.

**Gereç ve Yöntemler:** Elli beş AML hastasında NPM1 gen taraması için polimeraz zincir reaksiyonu ve tek-iplik konformasyon polimorfizmi kullanıldı.

**Bulgular:** Elli beş hastanın 12’sinde (%21,8) NPM1 mutasyonları bulundu ve mutasyon varlığının yüksek lökosit sayısı, kemik iliği blast oranı (sırasıyla, p=0,03; p=0,02) ve M5 alt tipi (p<0,001) ile anlamlı ilişkisi olduğu tespit edildi. NPM1 mutasyonu olan hastalar, daha yüksek oranda tam remisyon (p=0,003), daha düşük mortalite eğilimi, nüks ve dirençli hastalık riski (sırasıyla, p=0,28, p=0,45, p=0,08) taşımaktaydı. Sağkalım analizinde, hastalıksız sağkalım (18,635±1,229 vs. 11,041±1,250 ay, p=0,044) ve genel sağkalım (ortalama 19,810±1,624 vs. 12,063±1,244 ay, p=0,041), NPM1 mutasyonu taşıyanlarda taşımayanlara göre anlamlı olarak daha uzun bulundu. Çok-değişkenli analizde, NPM1 mutasyonunun hastalıksız sağkalım (HR=0,066, p=0,001) ve genel sağkalım (HR=0,125, p=0,002) için anlamlı bağımsız öngörücü olduğu görüldü.

**Sonuç:** NPM1 mutasyonu, Mısırlı hastalarda iyi gidiş ile ilişkili olan bir prognostik faktördür. NPM1 mutasyonu kullanılarak heterojen bir hastalık olan AML’nin prognoz açısından farklı bulunmuştur.

## INTRODUCTION

Acute myeloid leukemia (AML) is a heterogeneous group of clonal hematopoietic stem cell disorders. The development of AML is associated with accumulation of acquired genetic alterations and epigenetic changes in hematopoietic progenitor cells that alter normal mechanisms of cell growth, proliferation, and differentiation [1]. Molecular analyses have yielded novel gene mutations that serve important markers for prognostic stratification of the heterogeneous AML group. The nucleophosmin 1 (NPM1) gene is mapped to chromosome 5q35 and encodes a protein of 294 amino acids. Nucleophosmin protein (NPM), also known as B23, numatrin, and NO38, is an abundant nucleolar phosphoprotein constantly shuttling between the nucleus/ nucleolus and cytoplasm [2]. NPM1 may assist in ribosomal protein assembly [3] and maintain genomic stability through its participation in DNA repair [4]. It also plays a crucial role in cell cycle regulation and apoptosis via its interactions with tumor suppressor p53 and alternate reading frame protein. The NPM1 gene frequently acts as a target of chromosomal translocations and causes the cytoplasmic dislocation of proteins in various types of leukemia and lymphoma, indicating its role in malignant transformation [5,6]. Discovery of heterozygous mutations of the NPM1 gene, involving the C-terminus at exon 12, is apparently specific for AML as these mutations have not been found in other hematolymphoid malignancies [7]. Within the group of patients with AML who have a normal karyotype, various studies have shown that patients with NPM1-mutated AML had a complete remission rate similar to [8] or significantly higher than [9,10] patients with wild-type NPM1 AML. Most studies have shown a statistical trend toward favorable outcome in event-free survival and overall survival [10,11,12]. Further analyses in the context of other molecular aberrations have shown that patients with NPM1-mutated AML who have a normal karyotype and lack FLT3-ITD (representing about 30% of all AML cases) have a better prognosis. This also emphasizes the value of comprehensive molecular genetic screening, because screening may ultimately lead to improved risk stratification [13]. In the current study, we investigated a group of AML patients for NPM1 mutations to clarify the gene’s prognostic impact on disease outcome.

## MATERIALS AND METHODS

**Patients**

The present study included 55 newly diagnosed de novo adult AML patients representing various French- American-British (FAB) subtypes. They were 25 males and 30 females with mean age of 45.65±16.28 years. They were consecutive patients meeting selection criteria defined below. Written informed consent was obtained from the patients prior to their enrollment in this study. Patients were diagnosed according to standard diagnostic methods including cytomorphological, cytochemical, immunophenotypic (positivity by flow cytometry was defined as an expression in at least 20% of cells in the gated population of interest, compared to internal negative control cells), and cytogenetic evaluation. In addition, 10 healthy subjects with matched age and sex were selected to act as a control group. Bone marrow (BM) samples from patients with AML were subjected to Ficoll-Hypaque (Pharmacia LKB, Uppsala, Sweden) density gradient centrifugation. All samples taken at diagnosis were confirmed to contain more than 90% leukemia cells after enrichment by centrifugation. Inclusion criteria were status as a newly diagnosed AML patient and no history of previous treatment. Exclusion criteria were status as a secondary AML patient, markedly impaired hepatic or renal functions, concurrent severe and/ or uncontrolled medical conditions (e.g., uncontrolled diabetes, infection, or hypertension), family history of hematological malignancies, and positivity for FLT3/ITD gene mutations by polymerase chain reaction (PCR).

**Treatment Protocol**

Patients received the standard ‘3+7’ induction chemotherapy protocol: doxorubicin (30 mg/m^2^ per day) for 3 days and cytarabine (100 mg/m^2^ per day as a continuous 24-hour intravenous infusion) for 7 days [14]. Patients with acute promyelocytic leukemia (M3) received all-trans retinoic acid plus anthracycline [15]. BM aspiration was done between 21 and 28 days after initiation of chemotherapy. Consolidation comprised 3 to 4 courses of high-dose cytosine arabinoside (3 g/m^2^ every 12 hours on days 1, 3, and 5; in total, 18 g/m^2^). Following this, patients were followed up once every 3 months with clinical examination and complete blood counts. A BM aspiration was done if there was any suggestion of relapse on clinical examination or peripheral smear.

**Screening of NPM1 Gene Mutation by PCR-SSCP**

**Figure 1:**
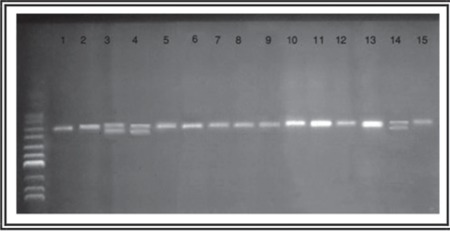
Agarose gel electrophoresis of NPM1 gene (exon 12): Lane 1 = Normal control sample. Lanes 2, 5, 6, 7, 8, 9, 10, 11, 12, 13, and 15 represent wild-type NPM1 gene. Lanes 3, 4, and 14 represent mutant NPM1 gene.

Genomic DNA was extracted from BM samples with the AxyPrep Blood Genomic DNA Miniprep Kit (Axygen Bioscience, USA) according to the manufacturer’s protocol. For NPM1 mutation analysis, NPM1 exon 12 was amplified by genomic PCR using primers NPMex12F/ CTGATGTCTATGAAGTGTTGTGGTTCC (sense) and NPMex12R/CTCTGCATTATAAAAAGGACAGCCAG (antisense). The reaction mixture was made up in a total volume of 50 μL by the following constituents: 100 ng of genomic DNA, 0.5 U Taq DNA polymerase, 1X Taq polymerase buffer, 1.75 mM MgCl2, 0.4 μM NPM1 primers, and 0.4 mM dNTP. The samples were amplified by initial denaturation at 95 °C for 5 min, followed by 35 cycles of 94 °C for 30 s, 53 °C for 1 min, and 72 °C for 2 min, and final extension at 72 °C for 10 min. They were checked on 2% agarose gel electrophoresis using a DNA marker (Figure 1).

**Figure 2:**
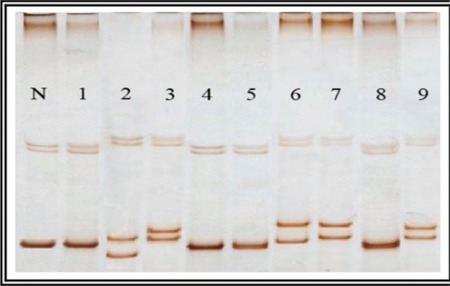
SSCP of NPM1 gene (exon 12): N = Normal control sample. Lanes 1, 4, 5, and 8 represent wild-type NPM1 gene. Lanes 2, 3, 6, 7, and 9 represent mutant NPM1 gene.

PCR products were mixed with 10 volumes of loading buffer and denatured at 96 °C for 5 min, quenched on ice immediately, and applied to 10% polyacrylamide gel electrophoresis. Normal NPM1 exhibits a specific conformational pattern. A mutant gene displays a pattern with different electrophoretic mobility (mobility shift) [16] (Figure 2).

**Criteria of Response and Survival Definitions**

Complete remission (CR) required bone marrow blasts of <5.0%, absence of blasts with Auer rods, absence of extramedullary disease, absolute neutrophil count of >1.0x 10^9^/L, and platelet count of >100x10^9^/L with independence from red cell transfusions. Relapse was defined by bone marrow blasts of ≥5.0%, reappearance of blasts in the blood, or development of extramedullary disease. Resistant disease was defined as more than 15.0% BM blasts after induction therapy. Overall survival (OS) was defined as the time from entry to death. For patients achieving first CR, disease-free survival (DFS) was defined as the time from first CR to an event (death in CR or relapse).

**Statistical Analysis**

Comparisons between data were performed with the Mann-Whitney test for continuous variables and the Fisher exact test for categorical variables. Survival curves for OS and DFS were calculated according to the Kaplan-Meier method and were compared using a 2-sided log rank test. Prediction of OS and DFS was done using multivariate analysis applying age, cytogenetics, FAB subtypes, total leukocyte count (TLC), and NPM1 mutation state as covariates. P<0.05 was considered statistically significant. SPSS 16.0 (SPSS Inc., Chicago, IL, USA) was used for statistical analysis.

## RESULTS

**Frequency of NPM1 Mutations**

PCR products from 55 newly diagnosed AML patients were used to screen for the prevalence of NPM1 mutation in exon 12. The wild-type gene was 167 bp. NPM1 mutation showed a double band at positions of 167 and 171 bp (Figure 1) in 12 of 55 AML cases (21.8%). All healthy control subjects had the wild-type allele.

**Gene Mutations and Clinical Characteristics**

All 55 patients were evaluated for clinical characteristics. The presence of NPM1 mutations was not related to age, sex, or the occurrence of fever, pallor, bleeding tendency, splenomegaly, hepatomegaly, or lymphadenopathy (p=0.39, 0.76, 0.516, 0.605, 0.217, 0.190, 0.416, and 0.230, respectively) (Table 1). TLC and BM blast cells were significantly higher in the NPM1 mutation group than in the wild-type group (p= 0.03 and 0.02, respectively) (Table 2). NPM1 mutations were significantly higher in FAB M5 (50%) than in other FAB subgroups (p<0.001) with a statistically insignificant difference between mutant and wild-type NPM1 regarding to FAB subtypes (p=0.65) (Table 2).

Cytogenetic data were available for all patients: 32 patients had normal karyotype while 7 had t(8;21)(q22;q22), 7 had inv16(p13.1q22), 4 had t(15;17)(q22;q12), and 5 had 11q23 abnormalities. According to the cytogenetics, NPM1 mutation was preferentially found in AML-NK patients (8 of 12; 66.7%). Among AML with abnormal cytogenetics, 2 cases with inv(16), 1 case with t(15;17), and 1 case with t(8;21) showed NPM1 mutations, with nonsignificant difference between mutant and wild-type NPM1 regarding to cytogenetics (p=0.509) (Table 1).

**Prognostic Impact of NPM1**

**Figure 3:**
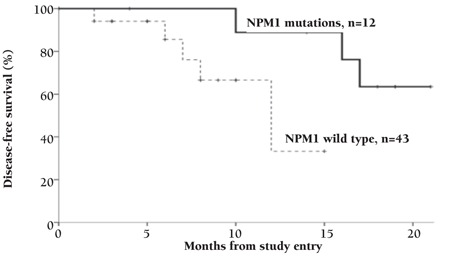
Kaplan-Meier curve for disease-free survival in wild and mutant NPM1 patients.

**Figure 4:**
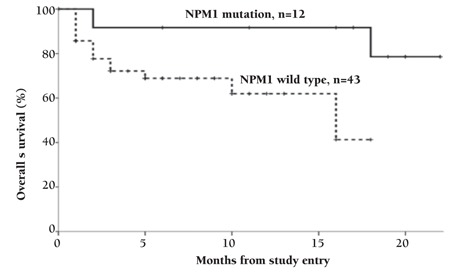
Kaplan-Meier curve for overall survival in wild and mutant NPM1 patients.

Twenty-four AML patients achieved CR after induction chemotherapy (43.6%). The CR rate was significantly higher in the patients with NPM1 mutations (9 of 12; 75%, p=0.003) than those without (15 of 43; 34.9%) (Table 3). Refractory disease, relapse, and mortality rate were nonsignificantly lower in cases of NPM1 mutation (p=0.08, p=0.45, p=0.28) (Table 3). Patients with NPM1 mutations had a significantly longer DFS (mean 18.635±1.229 versus 11.041±1.250 months, p=0.044) and OS (19.810±1.624 versus 12.063±1.244 months, p=0.041) than those without (Figures 3 and 4). Multivariable analyses confirmed NPM1 mutation as a significant independent predictor for DFS and OS (hazard ratio=0.066, p=0.001; hazard ratio=0.125, p=0.002, respectively) (Table 4).

## DISCUSSION

The prognostic effect of various chromosomal aberrations in AML is well established with implications for therapy. We evaluated the prevalence and prognostic impact of NPM1 mutations in adult AML patients. The incidence of NPM1 mutations was 21.8%, which was obviously lower than previously reported (35%, 45%, 64%) by some earlier studies [7,8,9] but approximately similar to that reported (25.8%, 28.2%) by others [10,17]. The lower detection rate may be due to a higher background of wild-type allele or lower percentage of NPM1 mutation-positive cells in some cases, in addition to variable numbers of cases, and some former studies also focused on NK-AML. Certain associations between gene mutations and clinical characteristics have been reported in the past years [18].

No significant differences were found between mutant NPM1 and wild-type patients regarding to age and sex. This is in accordance with the findings of other studies [10,19]. However, Suzuki et al. found that patients with NPM1 mutations were significantly older than those without mutations [10].

We also found a significantly increased TLC and blast cell percentage in patients with NPM1 mutations (p=0.03 and 0.02, respectively). This finding was consistent with some previous reports [10,13,20]. Boonthimat et al. reported that NPM1 mutations were particularly associated with higher platelet count [21], as was similarly observed by Thiede et al., who suggested that blasts with NPM1 mutation might retain a certain capacity for thrombocytic differentiation as demonstrated by in vitro experiments [22]. Regarding FAB classification, this study, in accordance with others, confirmed that NPM1 mutations occur most commonly in FAB class M5 (p=0.001) [7,13,20,23]. Association of NPM1 mutation with monocytic features of AML indicated a participation of NPM1 mutation in inducing leukemic development towards monocytic features. Cytogenetic data were available for all patients. The NPM1 mutation was nonsignificantly higher in patients with a normal karyotype (66.7%). Other studies showed significantly higher NPM1 mutations in AML-NK [7,8,24].

We were interested in the impact of NPM1 mutations on the hematological response following induction therapy as well as on the survival of Egyptian AML patients. For this reason, we considered the comprehensive data demonstrating that in AML patients an additional FLT3- ITD mutation is associated with a worse outcome compared with the expression of a single NPM1 mutation that can improve the prognosis in these patients [9,22]. We therefore excluded those patients harboring FLT3-ITD mutation from this analysis. The clinical outcome of NPM1 mutations (NPM+/FLT3-ITD-) seems to be distinctly favorable. The frequency of CR was higher (p=0.003) and mortality rate, relapse, and refractory disease were nonsignificantly lower (p=0.28, p=0.45, p=0.08) in patients with NPM1 mutations than those without. Other studies reported higher rates of CR in mutant NPM1 patients, suggesting that these patients are more sensitive to chemotherapeutic agents [12,25]. These studies assumed that NPMc+ may interact with and sequester nuclear factor kappaB, contributing to the maintenance and survival of malignant clones and an impaired response to chemotherapy in the cytoplasm, thus leading to its inactivation and reduced DNA binding [12,25]. However, in other studies, patients younger than 60 years old and pediatric patients did not show a significantly different CR rate between mutant and wild NPM1 [26,27]. DFS and OS were significantly longer for patients with NPM1 mutations. Similar results were also obtained in other studies [9,12,13,21,23] that demonstrated a favorable impact of NPM1 mutations on outcome. On the other hand, Boonthimat et al. reported that they did not observe a major difference in the OS in Thai patients with and without NPM1 mutation [21]. This contradiction may be attributed to ethnic variations, different inclusion criteria, and different sample sizes. Multivariable analyses confirmed NPM1 mutation as a significant independent predictor for DFS (hazard ratio=0.066, p=0.001) and OS (hazard ratio=0.125, p=0.002). Similar results were obtained in other studies [9,10], where NPM1 mutations were a favorable prognostic factor for DFS and OS. Other genomic abnormalities may accompany NPM1 mutations and have a prognostic impact in the NPM1-mutated patients, e.g., AML-NK patients without FLT3-ITD mutations and with both mutant NPM1 and isocitrate dehydrogenase (IDH) represent a favorable- risk subset defined by a specific mutational genotype, whereas patients negative for FLT3-ITD mutations who had mutant NPM1 without concurrent IDH mutations had a much less favorable outcome, particularly if those patients had concurrent mutations associated with an unfavorable- risk profile [28]. Schneider et al. showed that the FLT3-ITD mRNA level has a high prognostic impact in NPM1-mutated AML-NK, and that it contributes to relapse risk stratification and might help to guide postremission therapy in NPM1- mutated AML [29]. This is of major clinical importance, since it strongly suggests that NPM1 mutations may allow dissection of the heterogeneous group of AML into prognostically different subgroups. Since NPM1-mutated AML was listed as a provisional entity in the 2008 World Health Organization classification, routine screening of NPM1 gene mutations will eventually be needed to stratify patients with AML in the context of comprehensive genetic analysis. In conclusion, we propose that NPM1 mutations have prognostic significance in Egyptian AML patients; molecular assessment of NPM1 mutation at diagnosis offers valuable additional prognostic information and may thereby markedly affect therapeutic decisions.

**Conflict of Interest Statement**

The authors of this paper have no conflicts of interest, including specific financial interests, relationships, and/or affiliations relevant to the subject matter or materials included.

## Figures and Tables

**Table 1:**
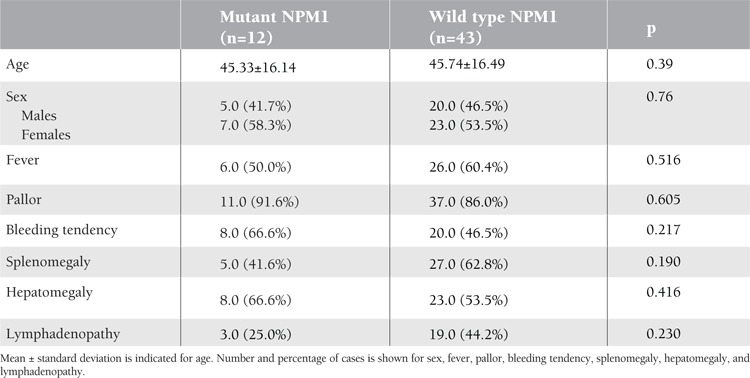
Clinical data of NPM1 mutations versus wild type in AML patients.

**Table 2:**
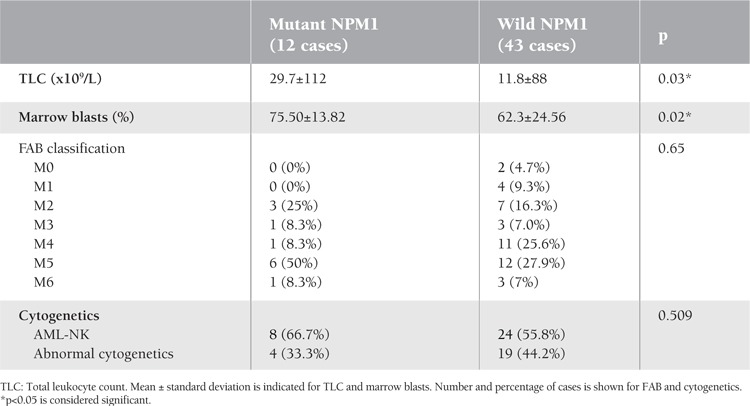
Laboratory data of NPM1 mutations versus wild type in AML patients.

**Table 3:**
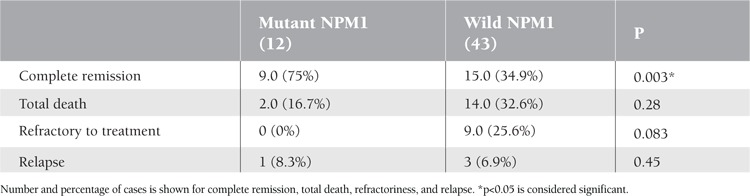
Comparison between positive NPM1 and negative NPM1 cases as regards clinical outcome.

**Table 4:**
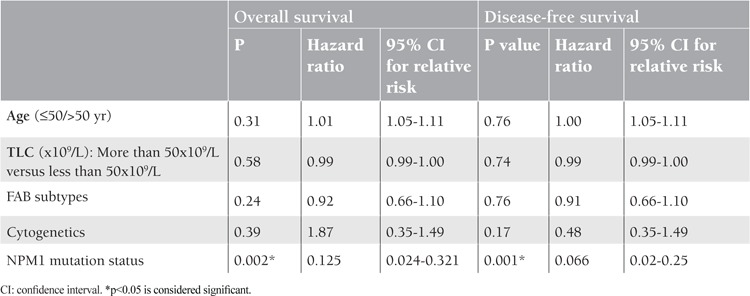
Overall survival and disease-free survival as dependent parameters studied with other covariates (multivariate analysis).
